# The risk of footswitch misstep during robot-assisted thoracoscopic surgery: a case report

**DOI:** 10.1093/jscr/rjab292

**Published:** 2021-12-14

**Authors:** Masayuki Hashimoto, Satoru Sawai, Mayumi Oshio, Makoto Yoden, Keiko Takeda

**Affiliations:** Division of Thoracic Surgery, Center for Respiratory Diseases, National Hospital Organization Kyoto Medical Center, Kyoto, Japan; Division of Thoracic Surgery, Center for Respiratory Diseases, National Hospital Organization Kyoto Medical Center, Kyoto, Japan; Department of Thoracic Surgery, National Hospital Organization Minami Kyoto Hospital, Kyoto, Japan; Division of Thoracic Surgery, Center for Respiratory Diseases, National Hospital Organization Kyoto Medical Center, Kyoto, Japan; Division of Thoracic Surgery, Center for Respiratory Diseases, National Hospital Organization Kyoto Medical Center, Kyoto, Japan

## Abstract

The Da Vinci Surgical System is an ergonomically devised and excellent surgical support device. However, surgeon skill is of paramount importance since human error cannot be completely eliminated. We report a case of bleeding from the pulmonary artery due to a footswitch misstep. A 72-year-old male with suspected right upper lobe lung cancer underwent robot-assisted thoracoscopic surgery (RATS). While avoiding the pulmonary artery with the right arm spatula and trying to cauterize V2t with the left arm bipolar-forceps, the footswitch was accidently activated and the spatula was energized, resulting in pulmonary artery trauma and blood loss. After this case, we changed the surgical procedure from a monopolar–bipolar combination use to a bipolar-only use and noted no significant difference in the console duration, and less intraoperative blood loss. Human errors can occur anytime. Especially for surgeons new to RATS, simplified foot management should be considered until RATS mastery is achieved.

## INTRODUCTION

The Da Vinci Surgical System (DSS) is a surgical support device that has been ergonomically devised and enables intuitive operability. This delicate instrument, with excellent mobility, allows the execution of fine surgical procedures with high-definition three-dimensional (3D) images and less hand shaking [1]. However, even with excellent surgical instruments, physician skill is of paramount importance [2] since human error cannot yet be completely eliminated [3]. We report a case of bleeding from the pulmonary artery due to a footswitch misstep.

## CASE REPORT

A 72-year-old male with suspected right upper lobe lung cancer (cT1aN0M0 stage1A1) underwent robot-assisted thoracoscopic surgery (RATS). Since his diagnosis of lung adenocarcinoma was obtained by diagnostic partial lung resection, we performed a RATS upper right lobectomy with lymph node dissection. While avoiding the pulmonary artery with the right arm permanent cautery spatula, and trying to cauterize V2t with the left arm fenestrated bipolar-forceps, the footswitch was accidently activated and the spatula energized. This resulted in trauma and pulmonary artery bleeding (Supplementary material, Video 1). Because the bleeding was stopped by compressing the pulmonary artery with surrounding tissue, the operation was continued without a thoracotomy conversion. After this case, we changed the surgical procedure from a monopolar–bipolar combination use to a bipolar-only use (Supplementary material, Video 2). For all cases by a single operator (M.H.) that used the X system from July 2019 to April 2020, we examined console duration, intraoperative blood loss, number of staples used, use of a vessel sealer and drainage duration before and after the surgical procedures (Table 1). We found no significant difference in the duration of console between the two surgical procedures. There was less intraoperative blood loss after the change. Acquisition of approval from our institutional ethics committee was not required because no additional personal information was revealed for this case report. Nonetheless, the patient in this case report provided written informed consent.

**Table 1:** Clinical features of monopolar–bipolar combination use/ bipolar-only use groups

	M–B group (*n* = 7)	B group (*n* = 11)	*P* value
Age (year)	72.5 ± 8.7	68.7 ± 8.6	N.S.^*^
Sex (male/female)	5 / 2	8 / 3	N.S.^**^
BMI (kg/m2)	24.0 ± 2.7	22.9 ± 3.1	N.S.^*^
COPD (no/yes)	5 / 2	5 / 6	N.S.^**^
p-stage (1A/1B/2A/2B)	5 / 1 / 0 / 0	5 / 2 / 1 / 3	N.S.^**^
Console duration (min)	150 (134–202)	147 (93–223)	N.S.^***^
Blood loss (ml)	10 (0–160)	0 (0–100)	<0.05^***^
Number of staples use	7.0 (4–9)	6.0 (6–8)	N.S.^***^
Use of a vessel sealer (no/yes)	2 / 5	7 / 4	N.S.^**^
Drainage duration (day)	2.1 ± 1.9	1.5 ± 0.7	N.S.^*^

M–B group: monopolar–bipolar combination use group.

B group: bipolar-only use group.

^*^Student *t*-test

^**^χ^2^ test

^***^Mann–Whitney *U*-test

## DISCUSSION

Misapplication of accelerators and brake pedals has been attracting attention in Japan, particularly as this issue relates to automobile accidents in the elderly. These efforts have revealed a peak in the under-24 age group, attributable to a lack of attention and experience and familiarity with automobiles [4]. This was the 14th case of RATS lobectomy, and at the time of the seventh case, our facility updated to the da Vinci X system. Thus, these results may reflect both a lack RATS experiences, in addition to unfamiliarity with the new system. RATS requires excellent operability of about 20 lobectomies to complete the learning curve, and ~60 to achieve mastery [5]. Therefore, it is expected that more experience with RATS will reduce mistakes, but it is difficult to avoid mistakes completely.

The DSS is equipped with Smart Pedal Technology. Here, the right and left arms are automatically associated with each pedal, thus preventing the reversal of left and right settings. When the foot sensors on either footswitch pedal panel detect a foot, a green line is displayed on the outer frame of the monitor to attract the surgeon’s attention. However—for example—if the toe is moved left and right without moving the heel, the foot sensor recognition may fail. It is also possible that the foot may accidentally touch the next pedal because there is no threshold between the left and right pedals. In order to avoid complicated foot management, we changed to bipolar-only use; however, there was no bleeding due to missteps and no disadvantages due to changes in the surgical procedure. It is not the only answer, the measures should be decided for each facility because the DSS allows us to select and combine various instruments. However, surgeons new to RATS should consider simplifying the procedure until they achieve mastery. We also expect that suppliers will develop additional tools to help prevent human surgical procedure errors.

## Supplementary Material

Video1_rjab292

Video2_rjab292
